# “Your status cannot hinder you”: the importance of resilience among adolescents engaged in HIV care in Kenya

**DOI:** 10.1186/s12889-022-13677-w

**Published:** 2022-06-30

**Authors:** Casey Adams, Millicent Kiruki, Robinson Karuga, Lilian Otiso, Susan M. Graham, Kristin M. Beima-Sofie

**Affiliations:** 1 Department of Global Health, University of Washington, Seattle, WA USA; 2 Department of Social Work, University of Washington, Seattle, WA USA; 3 Department of Research and Strategic Information, LVCT Health, Nairobi, Kenya; 4 Department of Medicine, University of Washington, Seattle, WA USA; 5 Department of Epidemiology, University of Washington, Seattle, WA USA

**Keywords:** HIV, AIDS, Adolescents, Resilience, Stigma, Kenya, Adolescents living with HIV

## Abstract

**Background:**

Approximately 40% of the 110,000 adolescents living with HIV (ALHIV) in Kenya have not achieved viral suppression. Despite the increasing availability of adolescent-friendly services, adolescents face barriers that impact ART adherence. This study aimed to identify key stigma-related barriers to ART adherence and strategies used by adolescents in overcoming these barriers.

**Methods:**

Data were collected by LVCT Health, a Kenyan organization with a programmatic focus on HIV testing, prevention, and care. 122 participants were recruited from 3 clinical sites affiliated with LVCT Health in Nairobi, Kisumu, and Mombasa. In-depth interviews were conducted with ALHIV (*n* = 12). Focus group discussions were conducted with ALHIV (*n* = 5), peer leaders (*n* = 3), and adolescents receiving HIV services in community settings (AIC) irrespective of HIV status (*n* = 3). Interviews and focus groups were audio recorded, translated, and transcribed. Data were analyzed thematically, with a focus on stigma and resilience.

**Results:**

While AIC primarily focused on adherence barriers and stigma, ALHIV and, to some extent, peer leaders, also identified resilience factors that helped overcome stigma. Four major themes emerged: 1) knowledge and future-oriented goals can drive motivation for ALHIV to remain healthy; 2) disclosure to others strengthens support systems for ALHIV; 3) medication-taking strategies and strategic disclosure can overcome adherence challenges in school; and 4) a supportive clinic environment promotes continuous adolescent engagement in HIV care. These concepts were used to develop a conceptual stigma/resilience model depicting how resilience moderates negative effects of stigma among ALHIV.

**Conclusions:**

This study demonstrates the positive effects of ALHIV resilience on ART adherence and illuminates how stigma impacts ALHIV differently depending on their resilience. Strengths-based interventions, focused on increasing resilience among ALHIV in Kenya, and more formal involvement of adolescent peers to bolster adolescent support, have the potential to improve ART adherence among ALHIV.

**Supplementary Information:**

The online version contains supplementary material available at 10.1186/s12889-022-13677-w.

## Background

Adolescents represent a highly vulnerable age group in the context of the human immunodeficiency virus (HIV) epidemic and constitute a growing proportion of people living with HIV globally [1]. Almost 90 percent (approximately 1.5 million) of all adolescents living with HIV (ALHIV, 10–19 years of age) reside in sub-Saharan Africa, the region with the highest burden of HIV globally [2]. Kenya has the fourth-largest HIV epidemic [3], with an HIV prevalence among adolescents of approximately 0.9% (95% confidence interval [CI]: 0.6%–1.3%) [3, 4]. In Kenya, adolescents and young adults (AYA) are the age group at the highest risk for dropping out of HIV treatment, which increases their risk for adverse health consequences, including virologic failure and death [4, 5]

Adolescence is a time of physical growth and sexual maturation when individuals struggle to navigate relationships and make independent decisions [6, 7]. ALHIV face challenges living with a chronic disease while transitioning from childhood to adulthood. In addition, misinformation, ignorance, prejudice, and discrimination can lead to HIV-related stigma, a widely recognized barrier to positive health outcomes among ALHIV [8–10]. Stigma drives social responses such as rejection and isolation that ALHIV experience in multifaceted ways, including internalized, anticipated, and enacted stigma [11]. Moreover, adolescents may experience stigma differently in multiple contexts. Some adolescents may experience discrimination from students at school but have a supportive home environment. In contrast, others may feel rejected by family members but receive support from friends or counselors. Stigmatization can adversely affect adolescents’ mental health, discourage disclosing HIV status, impede access to social support systems, and exacerbate challenges with antiretroviral treatment (ART) adherence and engagement in health services [8, 12].

Understanding the sources and drivers of stigma can lead to a better awareness of how adolescents strive to overcome these barriers through resilience. Although resilience theory has been less widely cited in the ALHIV literature than stigma, recent research has explored how healthy outcomes can occur in the presence of risk factors [13]. Despite the harmful effects of stigma, many ALHIV find strength through social networks and internal resilience, which can help counteract the adverse effects of stigma [14]. This resilience can take many forms and is present in many contexts. Thus, identifying key barriers to ART adherence and adolescents’ resilience in overcoming these barriers can inform the development of interventions directly responsive to ALHIV needs and harness identified resilience strategies.

The desire to address disparities in ALHIV outcomes and provide adolescent-friendly services has driven the development of strategies to improve health outcomes among ALHIV. A random sample of 102 HIV clinics in Kenya examined current strategies used by clinics to improve ALHIV adherence and retention [15]. The study found that common strategies included offering adolescent support groups (97%), dedicating specific clinic days to adolescents (91%), and offering clinic days on weekends (57%). There have also been heightened efforts to hire staff from whom adolescents may feel more comfortable receiving care and counseling, as reflected in the vast majority (89%) of clinics that currently utilize peer leaders (also referred to in the literature as “peer educators”) [15]. While many clinics offer adolescent-friendly services, few studies have evaluated the impact of these services on ALHIV outcomes or addressed care engagement barriers outside clinic settings. A recent systematic review examining interventions to meet the needs of ALHIV in low and middle-income countries, mostly from sub-Saharan Africa, found a significant gap in evidence supporting interventions that improve outcomes along the ALHIV care continuum [16]. To address this evidence gap, we aimed to better understand how stigma impacts adolescents’ ART experiences across different contexts, from the perspective of ALHIV, adolescents in the community, and peer leaders. Using a socio-ecological model, we evaluated barriers and facilitators to ART adherence and care engagement, and identified strategies for how ALHIV cope with stigma using resilience.

## Methods

### Study design

We conducted a qualitative analysis of data collected for the Most at Risk Adolescent Research Project led by LVCT Health. LVCT Health is a Kenyan non-governmental organization that provides HIV testing, HIV prevention and care and treatment, sexual and reproductive health (SRH), and gender-based violence services targeting adolescents, young people, and other vulnerable populations [17]. LVCT Health researchers conducted in-depth interviews (IDIs) and focus group discussions (FGDs) with three stakeholder groups: 1) ALHIV receiving clinical care from LVCT-supported sites, 2) adolescents who were beneficiaries of HIV information and services in community programming (AIC), irrespective of HIV status, and 3) AYA peer leaders who provided support to ALHIV through LVCT and partner clinics, irrespective of HIV status. LVCT Health selected the three participant groups to provide a holistic understanding of the facilitators and barriers to HIV services among ALHIV.

### Study population and setting

Participants were recruited from health facilities in three Kenyan counties with a high burden of HIV [18]: Kisumu, Nairobi, and Mombasa. Nairobi and Kisumu clinics were run by LVCT Health and provided HIV care and prevention services to all age groups. The Mombasa clinic, dubbed “Youth Zone,” was government-operated but supported by LVCT Health, and had services tailored to AYA. All three HIV clinics had ALHIV patients and peer leader programs to support ALHIV with ART adherence and retention in care. ALHIV were eligible if they were between 10 and 19 years old and had been receiving ART > 6 months. The study included a mix of ALHIV with good ART adherence (2018 Kenya ART guidelines guided definition: good adherence (viral load < 1,000 copies/mL), poor adherence (viral load ≥ 1,000 copies/mL)). AIC were eligible if they were between 15 and 19 years old and receiving HIV prevention services from LVCT Health’s youth HIV prevention programs (Kisumu and Mombasa) or other local HIV prevention programs (Nairobi), both of which represented non-clinical care. AIC’s were recruited from a non-clinical setting. They were receiving information and / or HIV prevention services from LVCT Health or local HIV programs. Peer leaders were eligible if they were between 20 and 24 years old and worked at one of the LVCT-affiliated study clinics; some worked at partner ALHIV programs in Nairobi due to low numbers attached to the study site.

### Data collection

IDIs and FGDs were conducted in 2018 using semi-structured interview guides (see Additional files 1, 2, 3 and 4) which focused on eliciting adolescents’ views and knowledge on four main topic areas, including: (1) experiences taking ART, (2) barriers to clinic attendance and ART adherence, (3) disclosure of HIV status, and (4) recommendations for how to improve ALHIV adherence and care engagement. Semi-structured interview guides did not include the terms “resilience” or “stigma”. Instead, moderators used open-ended probes to explore participants’ perspectives and experiences related to stigma and resilience concepts. Question phrasing was customized for each subpopulation to optimize relevance.

ALHIV participated in both IDIs and FGDs, to capture both depth of individual lived experiences and general perspectives from this group. ALHIV who participated in FGDs were members of the same clinic-based support groups and familiar with each other to avoid unintended disclosure. Peer leaders and AIC participated in FGDs designed to generate dynamic discussion about participants’ general perceptions of ALHIV experiences. Where possible, ALHIV and AIC FGDs were stratified by sex to enhance participant comfort during the discussion of themes related to SRH. One FGD in Mombasa was sex mixed. FGDs and IDIs were conducted in English, Luo, or Swahili, depending on participant preferences. Discussions were audio-recorded, transcribed verbatim in the interview language, and translated into English for analysis. IDIs lasted an average of 40 min. FGDs included 8–12 participants and lasted an average of 91 min.

### Data analysis

Data were analyzed using thematic analysis to identify key influences related to stigma and resilience on ART adherence and care engagement among ALHIV. A combination of three existing theoretical models informed analysis: the social-ecological model, the HIV stigma framework, and resilience theory. The socio-ecological model conceives of the ecological environment as a nested arrangement of systems, with four levels of influence: (1) structural factors, such as policy, laws, and infrastructure; (2) community factors, such as school settings and clinic services; (3) interpersonal factors, such as relationships with family and friends; and (4) individual factors, such as adolescents’ HIV-related knowledge, attitudes, and internalized stigma [19]. The HIV stigma framework characterizes how ALHIV experience HIV-related stigma through multi-level effects on adolescents’ thoughts and feelings, actions and behaviors, and sense of belonging or community [11]. Resilience theory explores the complex interactions between an individual’s assets and resources [20] and has been used in research with adolescents to explore how positive influencing factors (also referred to as “promotive factors”) operate in the presence of risk [21]. Low resilience is associated with poor outcomes due to stigmatization, while high resilience leads to resistance to stigma [22]. Drawing upon resilience theory, ALHIV resilience was operationalized during data analysis as the process of overcoming barriers to ART adherence or clinic attendance, coping successfully with setbacks including HIV stigma, and maintaining motivation to succeed and avoid a negative trajectory for health and overall well-being [14].

Informed by these theoretical models, the research team developed a continually updated and revised codebook throughout the coding process. CA and MK each coded half the transcripts in Dedoose (version 4.12, SocioCultural Research Consultants, LLC). All coded transcripts were then reviewed by another member of the team. For each participant group, queries were used to extract coded quotations, which were reviewed and summarized, and grouped into themes by level of the socio-ecological model (i.e., individual, interpersonal, community, structural). Themes were compared between participant groups and socio-ecological levels to gain a macro-level understanding of the similarities and differences between and across levels and populations.

### Ethical approval

Ethical approval and oversight of data collection were provided by the AMREF Ethics and Scientific Review Committee (#P396/2017). The University of Washington Institutional Review Board provided an exempt determination for data analysis (#00,013,543). All participants provided written informed consent (if 18 years or older) or assent (for those below 18 years). Written parental consent was obtained for those below 18 years.

## Results

A total of 122 AYA participated in 11 FGDs and 12 IDIs: 38% from Nairobi (*n* = 46), 37% from Kisumu (*n* = 45), and 25% from Mombasa (*n* = 31). Participant ages ranged from 15–24 years, and 57% were female (*n* = 70). Additional demographic details are presented in Table 1.

**Table 1:** Participant characteristics

Characteristic	Total	FGDs			IDIs
		**ALHIV**	**AIC**	**Peer Leaders**	**ALHIV**
Total participants	122	49	30	31	12
Female ^*^	70 (57)	26 (53)	22 (73)	16 (52)	6 (50)
Age range	15–24	15–19	15–19	20–24	15–19
Location ^*^					
Nairobi	46 (38)	19 (39)	11 (37)	12 (39)	4 (33)
Mombasa	31 (25)	11 (22)	8 (26)	8 (26)	4 (33)
Kisumu	45 (37)	19 (39)	11 (37)	11 (35)	4 (33)

^*^ n (%)

Adolescents described how multiple stigma-related barriers and resilience-related facilitators, occurring at individual, interpersonal, and community levels, influenced ALHIV adherence to ART (Table 2). While AIC and peer leader participants mainly focused on barriers to adherence, ALHIV adopted a strengths-based perspective. ALHIV described how they had developed resilience independently and through support systems, and how this resilience manifests in better adherence to ART and clinic attendance. Overall, we identified four major themes that describe the interplay between stigma and resilience among ALHIV. Within each thematic area, resilience was a significant theme throughout discussions with ALHIV. However, peer leader and AIC participants were less likely to recognize resilience and its contribution to stigma reduction. Peer leaders and AIC easily identified challenges that ALHIV faced, but most did not discuss how ALHIV can overcome barriers to achieve ART adherence.

**Table 2:** Perceptions of barriers and facilitators to adolescent ART adherence by participant group

Socio-ecological model level	Barrier	ALHIV	AIC	Peer leader	Facilitator	ALHIV	AIC	Peer leader
Individual	ALHIV experience internalized stigma, which manifests in isolation and a lack of sense of belonging	X	X	X	Self-motivation and a positive attitude drive pro-health behaviors	X		
	Internalized stigma leads to low morale, negatively impacting ART use	X	X	X	The presence of future life goals (i.e., career goals, family goals) is a motivator for adherence	X		
	ALHIV constantly compare themselves to peers without HIV, negatively impacting mental health	X			Knowledge is power, and accurate knowledge of ART can improve adherence	X		X
Interpersonal	Many parents/families will stigmatize, mistreat, or desert ALHIV when they find out their status		X	X	Family members offer support in the form of encouragement, reminders to take ART, and help picking up medication	X		
	Stigmatization within families often leads to community-wide stigma		X	X	Friends are generally supportive when disclosure occurs; support includes encouragement and reminders to take ART	X		
	ALHIV struggle with disclosing their status due to anticipated stigma	X	X	X	Support of an HIV-positive friend or family member encourages better adherence and improves mental health	X		X
	Lack of disclosure to others can lead to social withdrawal and mental health concerns (i.e., anxiety, depression)	X			Having the support of even one person (family, friend) can motivate ALHIV to engage in pro-health behaviors	X		X
	ALHIV default on medication (skipping or delaying ART) due to fear of status disclosure while with friends or family	X	X	X	Disclosure is challenging, but the support received, as a result, is worth it	X		X
	Disclosure to friends causes friends to stigmatize/isolate ALHIV or become overly attentive		X					
Community	School classmates and teachers stigmatize ALHIV and spread gossip	X	X	X	ALHIV who seek support from school staff (headteachers, matrons, nurses) are granted permission to attend clinic appointments	X		
	ART medication packaging is stigmatizing (i.e., seen as a symbol of HIV) and discourages ALHIV from carrying pill bottles at school	X	X	X	In school settings, creative solutions for carrying ART discreetly facilitate adherence (ex: carrying single pills in pocket rather than pill bottle)	X		
	Teachers separate students with HIV from other students, causing social isolation for ALHIV in school	X	X	X	Positive relationships with HCW generate trust and encourage care-seeking among ALHIV	X		
	Distrust of teachers causes challenges asking for permission to attend clinic during school hours; this can lead to missed appointments	X			Peer leader encouragement and support is effective in motivating adherence, especially to attend clinic	X		X
	Fear of disclosure at school makes ART adherence difficult for those whose pill regimens overlap with school hours (especially boarding school students)	X	X	X	Adolescent support groups foster peer connection, support positive mental health, and improve adherence	X		X
	HCW are usually older, judgmental, cold, and unrelatable to ALHIV		X	X				
	HCW scold ALHIV who have missed appointments or have poor adherence, impacting their desire to attend clinic	X		X				

^This table outlines which participant groups discussed each of the above themes, to highlight each participant group’s perceptions of barriers and facilitators to ART adherence^

### Self-acceptance of HIV status and future-oriented goals can drive motivation for ALHIV to remain healthy

For many ALHIV, beginning ART was challenging, especially for those adolescents who had trouble accepting their positive HIV status. Initially, fear of unknown medication and potential side effects was daunting. However, with time, ALHIV noticed the positive effects of taking ART, which motivated them to continue medication to remain healthy.

*“First, before I began, I was very weak… But when I started taking the drugs, after some time, I saw some change in my skin…So I saw that the drugs were helping me…I got to know my status, and I decided: If this is what has been planned for me, I will continue to take the drugs and take care of myself.”*

‒ ALHIV IDI female, Mombasa.

Although it took time for many ALHIV to get used to their medication and new daily pill-taking routines, many noted that adherence became easier once they had adjusted to this change.

*“If you start and just continue, it will be easy. It will be just like taking tea; you will know this is the same formula every morning or your time. You will just get used to it. A journey starts like that and when you start, just finish your race.”*

‒ ALHIV FGD female, Nairobi

Despite adjusting to the routine, the majority of ALHIV described simultaneously experiencing internalized stigma, which negatively impacted mental health and lowered self-esteem. Constant comparisons to healthy peers, especially siblings or friends, led some to have difficulty accepting their status. Many adolescents who expressed mental health concerns and feelings of isolation also mentioned defaulting on medications.

Status acceptance was noted as a counterbalance to internalized stigma, with many ALHIV noting that consistent pill-taking became more manageable once they accepted their HIV status. ALHIV who accepted their HIV status trusted that they could continue to lead healthy lives with consistent ART adherence, and cited future aspirations, including educational, career, or family goals, as motivators for consistently taking ART.

*“I just take them [ART pills] very fast, go to sleep, and in the morning I wake up with a lot of psyche…When I go back to sleep, I take them again. It is very easy at that point when you have something that is driving you to live. When you have something that is pulling you towards life, it is easier than when there is not a lot happening; when things are at a standstill.”*

— ALHIV IDI male, Kisumu

ALHIV also recognized the importance of staying healthy for the benefit of loved ones, feeling a personal responsibility that also motivated adherence.

*“I think I’ll still take my meds because there’s still a lot that I need to do; there are still a lot of people that are depending on me, and there are still a lot of people that I need to show that life can go on after this.”*

— ALHIV IDI male, Kisumu

Throughout, a recurring theme was the importance of optimism and a positive outlook on life, which allowed adolescents to overcome internalized stigma and generated an internal motivation to stay healthy that facilitated ART adherence.

### Disclosure to family and other trusted individuals strengthens support systems for ALHIV

A significant theme throughout the data was fear of HIV status disclosure. At least one parent or caregiver was often aware of ALHIV status early for those with perinatal HIV acquisition. However, anticipated stigma made disclosure to others difficult, leading many to hide their status from all but their closest family members. Many ALHIV made conscious decisions to disclose their status to only a select few “critical people” who they were confident would be sources of support.

*“[Tell] that person the truth, but also when you are HIV positive, you can’t tell everyone. You just tell those people that you think if you tell this one there is a way he/she can help me and will not tell anybody else. In our family, it’s only four people who know. Those critical people, you show them that… it’s not the end of life, and you can make it in life.”*

‒ ALHIV IDI female, Mombasa

AIC and peer leaders particularly expressed concerns that family members and friends would stigmatize ALHIV if their HIV status were disclosed.

*“If you have it don’t go telling other people…if you do so it becomes a risk to you…the other people will start to isolate you and do bad things, refuse to play with you. [L]et’s say if you are in the same corridor when you are mopping, they can come and make it dirty because they are seeing you have HIV.”*

AIC FGD female, Nairobi

Despite this frequently endorsed fear, many ALHIV shared positive experiences with disclosure and described the support they received upon opening up to family or friends. ALHIV who had disclosed to their families noted that they were supported in their treatment adherence. Support, especially from mothers and aunts, was vital in driving resilience and motivating adherence; the role of fathers or male guardians was described less frequently. Support from family members was twofold: 1) emotional support, including encouraging them and boosting self-esteem, and 2) practical support, such as picking up their medications for them when they were unable to attend clinic or reminding them to take their ART on schedule.

*“If your parent really cares about you, your status cannot hinder you at all. She will encourage you to finish the medicine, and you will be okay. She will give you the medicine and give you encouragement, examples, teachings, and education.”*

‒ ALHIV FGD female, Nairobi

Friends' support varied widely and was mainly based on whether adolescents had disclosed their HIV status to friends. The vast majority of ALHIV recognized the value of disclosing to one or a select few “trustworthy” and “confidential” friends. Those who had done so described overwhelmingly positive responses to disclosing their status to friends. When disclosure did occur, friends helped adolescents adhere to ART by reminding them to take medications, keeping track of appointments, encouraging them, and providing emotional support.

*“So if you tell your friend on this particular day, she reminds you to go to the clinic. So your friend can set the alarm, or she can circle a calendar. So when that day comes, she tells you that you are supposed to go to the clinic, ‘Stop what you are doing. I’ll finish it, so you just go’.”*

— ALHIV FGD male, Kisumu

Some ALHIV had friends who were also HIV positive, which enabled mutual support. One ALHIV described a system she created with her friend in which they used certain code words to remind each other to take their ART, thus facilitating reminders in public without fear of inadvertent disclosure.

### Strategies for discrete pill-taking and strategic disclosure can overcome challenges at school

Adolescents spend much of their time in school, an environment that adolescents described as creating specific challenges for adherence. HIV stigma in school was noted as being extremely high and could lead to peer rejection, gossip, and “special treatment” from teachers that further isolated ALHIV from others.

*“The teachers are not on your side, nobody is on your side, students are isolating you, you cannot even reach your full potential so what exactly are you doing in school… So most resort to just dropping out…”*

-AIC FGD female, Nairobi

Often school staff, and teachers in particular, were viewed as untrustworthy and unsupportive. Most ALHIV had not disclosed their HIV status to teachers. However, teachers were described as being uninformed about HIV and lacking understanding of special needs and considerations for students living with HIV. Teachers were viewed as strict and unwavering in their school policies, which often prohibited students from carrying water or using the bathroom during class, therefore creating logistical barriers to adherence among adolescents whose pill schedules required them to take medication while in school.

ALHIV described overcoming these barriers by employing creative strategies for taking pills discreetly to avoid inadvertent status disclosure. For example, some adolescents carried pills in their pockets or socks rather than the noisy pill container, used plastic bags, or wrapped pills individually in paper. Many adolescents found ways to take pills during breaks, at the back of the classroom, or in the bathroom.

*“When I went to a boarding school…I was given the idea to use the school bell, I used to use the 8 am and 8 pm bell, the morning bell was for going to the assembly, and I had my medicine ready, I would at times pretend to my friends that I am taking water so that they wait for me then we run to the assembly. The evening bell was for bathing, so I would just take my medicine at ease.”*

‒ ALHIV FGD participant (unknown sex), Mombasa

Strict or inflexible school policies also created challenges for adolescents who required permission to miss class for clinic appointments. Many ALHIV stated that they had been denied permission to leave class, leading to missed appointments. To overcome these barriers, some ALHIV asked their parents to request permission from the school to attend clinic visits, and others obtained a note from the clinic or hospital to validate their request. ALHIV, who feared disclosure to staff members, sometimes resorted to missing the entire school day, later explaining to teachers that they had been sick.

Some ALHIV were able to build relationships with school staff they trusted to keep their status hidden and gain support for navigating adherence in the school setting. These school staff, most frequently school nurses or headteachers in day schools or matrons in boarding schools, supported ALHIV by allowing excused absences, storing medications, and reminding ALHIV to take their ART.

ART adherence barriers differed depending on the type of school ALHIV attended: boarding or day school. The majority’s opinion was that day schools improved adherence because they overcame the challenge of needing to hide medications while at school, as most ALHIV took medication before and after school. However, some adolescents attending boarding school felt that the routine, especially with consistent meal times, helped them maintain their ART schedule. In addition, those who felt comfortable disclosing their status to their roommates could take ART freely in their rooms, which facilitated adherence.

Regardless of the type of school they attended, ALHIV expressed a desire for schools to be more lenient in granting permission for absences. Peer leaders also recognized these barriers and some expressed a desire to attend schools to speak with students and staff with the goal of de-stigmatizing HIV and perhaps even affecting change in school policies.

### A supportive clinic environment promotes continuous adolescent engagement in HIV care

Most ALHIV felt supported by their health clinics and clinic staff. ALHIV and peer leaders agreed that youth-friendly services were essential for engaging adolescents in care and providing a sense of belonging and age-specific support. Various adolescent-friendly services discussed included youth-specific clinic days, youth-friendly clinic staff (including peer support), “youth zones” (youth-specific clinics), adolescent support groups, and treatment buddy systems (an arrangement in which two ALHIV mutually support each other for ART adherence through reminders, encouragement, and shared strategies). Most ALHIV valued youth-specific clinics or clinic days, which created a more comfortable atmosphere for adolescents, and most importantly, provided confidentiality.

*“You go to a place where you are comfortable; some of us are infected, and you don’t want your friends to know that you are infected. So you go to a place where it is safe; they [others] will not see you there.”*

‒ ALHIV FGD female, Kisumu

Youth-specific services also allowed adolescents to meet and socialize with others who shared their status and related to their challenges. In particular, adolescent support groups allowed adolescents to share personal experiences and work through problems in a safe space:

*“When we came here in the morning, we weren’t talking to each other because we don’t know each other. But [the healthcare worker] came and told us that we are the same, so we got the confidence; when I talk, I am ok, and when she talks, she is also ok. So the self-esteem and the confidence; you get encouraged, and you will be okay.”*

‒ ALHIV FGD female, Nairobi

ALHIV had largely positive experiences regarding their relationships with clinic healthcare workers (HCWs) and valued clinic staff who were understanding, confidential, non-judgmental, and supportive. They wanted clinic staff with whom they could feel free to ask questions and share concerns and who encouraged them and cheered them on. This type of support helped foster trust between HCWs and adolescents, even as many ALHIV were initially wary of opening up to HCWs.

*“I can say that what helped me a lot is the doctor, the good friend that I have… She keeps on encouraging me to move on and to maintain…in fact; she told me that so many people have the virus, so I’m not alone…so with that motivation, I was somewhat encouraged it was like a will that guides me to move on.”*

‒ ALHIV IDI male, Kisumu

Despite the overall positive experiences with HCWs described by ALHIV, peer leaders and AIC perceived most HCWs to be cold and unsympathetic to ALHIV struggles. Some ALHIV also felt that HCWs were too strict, and described situations where HCWs scolded adolescents who had missed appointments, causing them embarrassment and impacting their willingness to attend the clinic in the future.

An essential benefit of youth-specific clinics came from peer leaders, who ALHIV described as more relatable and trustworthy than other HCWs. Peer leader presence created a welcoming atmosphere in the clinic, where ALHIV were more likely to return and felt freer in discussing their questions and concerns. Some ALHIV expressed that support from peer leaders encouraged them to take ART consistently, helping them attain an undetectable viral load.

*“She [mom] would tell me to take [ART] by 8 am, but sometimes I would oversleep and miss it. So some of my bottles were full. So our counselor [the peer leader] encouraged me...told me what it [HIV] is and how to take my drugs, and since then, I am fine. My viral load came from 94k to 10k, and now it’s 150.”*

*‒* ALHIV FGD male, Nairobi

Peer leaders recognized their influence among adolescents and took this responsibility seriously.

*“[M]y role as a peer leader is that I can be able to influence the youths positively...if I go and influence or educate my fellow youth at least they can get something that they can apply and change their lives.”*

-Peer leader FGD participant (unknown sex), Kisumu

However, peer leaders also discussed a lack of adequate resources, compensation, and training to do their jobs effectively. They saw a more significant opportunity for positive intervention given sufficient resources and enhanced training. For example, peer leaders requested better education regarding ART, including side effects, to counteract common misinformation among adolescents.

*“[W]e should be provided with enough information in that even if I go to talk with people I will have that confidence of knowing what I’m talking about…if you have adequate information that you can tell someone the correct thing; the correct information.”*

-Peer leader FGD participant (unknown sex), Kisumu

In addition, peer leaders suggested that increased home visits would help adolescents struggling with challenges such as denial of their HIV status, fear of disclosure, and internalized stigma. Though outside their traditional scope of work within clinics, peer leaders expressed a desire to work within schools as well. Though potentially logistically challenging, peer leaders felt they could engage with school staff to enhance the support provided to students living with HIV and engage with students to provide education and counteract stigma in the school setting. Most peer leaders overlooked adolescents’ resilience and strength in overcoming adherence barriers and dealing with adversity. However, peer leaders were passionate about their role in working with ALHIV and expressed a willingness and enthusiasm to learn more and establish deeper relationships with ALHIV.

### Conceptual model

**Fig. 1:**
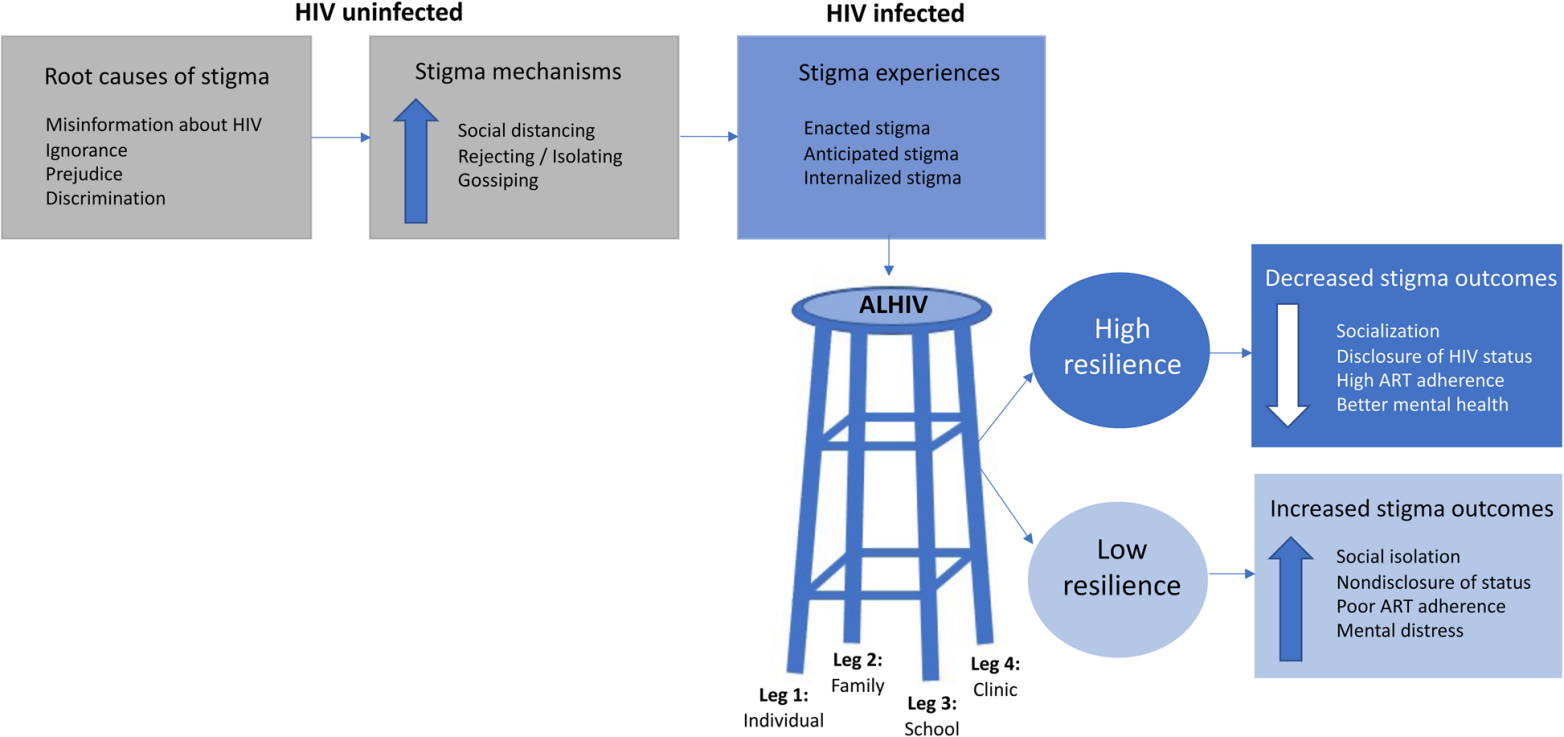
Conceptual model. This model depicts the interaction of stigma and resilience among ALHIV in Kenya. Root causes of stigma lead to manifestations of stigma in key contexts—represented by the four legs of a stool. Note that each leg of the stool is connected by reinforcing bars, representing the interconnection of the four themes. If any one leg is missing or becomes weak, it negatively influences the overall stability of the stool, reflecting the need to take a holistic approach for addressing root causes of stigma. ALHIV may respond to stigma differently, leading to different health outcomes

Our thematic analysis led to the development of a conceptual model (Fig. 1) that describes how stigma and resilience interact among ALHIV accessing LVCT Health services in Kenya. The model explores how, under certain conditions (e.g., a non-supportive family environment or stigma at school), stigmatization by HIV uninfected individuals may operate to produce adverse outcomes among ALHIV. However, adolescents identified that promotive factors could moderate the adverse effects of stigmatization. The four themes discussed above are represented as four legs of a stool, and represent influencing factors in different areas of adolescents’ lives, in which ALHIV can overcome stigma and build resilience. The four themes are interdependent, and presence or absence of any one can influence the others. This analogy demonstrates how ALHIV adherence is most robust when adolescents have positive influences in not one, but all four aspects of their lives: individually, from friends and family, at school, and in the clinic. For example, when adolescents possess self-motivation and are supported in their schools and health clinics and by family and friends, this can build a strong foundation for leading healthy lives. However, the presence of negative influencing factors in any one of these four contexts may upset the balance and frustrate efforts for ALHIV to achieve healthy adherence outcomes.

## Discussion

This qualitative analysis of adolescent experiences allowed for a better understanding of how adolescents, their support systems, and their communities can address stigma and support resilience. Our study highlighted how adolescents struggle with ART adherence and care engagement due to limitations of resilience’s moderating effects. This study informed the development of a conceptual model describing how resilience moderates the adverse effects of stigma among ALHIV, and highlighting the importance of support and resilience in four critical areas of the ALHIV context (i.e., individual, family and friends, school, and clinic).

The socio-ecological model has been used frequently to illustrate how social influences impact HIV outcomes, particularly in settings with widespread stigma [14, 23, 24]. The socio-ecological model informed our analysis by expanding our focus past individual-level challenges to incorporate challenges in different contexts such as the home, school, and clinic environments. Our conceptual model displays the different contexts by utilizing the stool analogy to convey how supportive environments at different levels of the socio-ecological model are linked together and support in all levels is vital to achieving positive health outcomes. Together, the HIV stigma model and resilience theory coupled with the socio-ecological model allowed for a unique, strengths-based approach to better understand how to harness adolescent strengths at various levels of the socio-ecological model to improve ART adherence and care engagement.

Though the association between stigma and resilience has been studied infrequently among ALHIV in Africa, one recent study recruited 385 adolescent participants ages 13–18 with HIV in South Africa to measure the relationship between stigma and resilience using the Child and Youth Resilience Measure [25]. The study found a negative relationship between HIV-related stigma and adolescent resilience, and that higher resilience was correlated with viral suppression [25]. Qualitatively, our findings illustrate that adolescent resilience, or a lack thereof, can lead to significant differences in health outcomes, as depicted in our conceptual model. Stigma can operate in different contexts (e.g., at home, in school) to adversely impact adolescents’ health outcomes if they cannot find support through various resilience strategies. On the one hand, ALHIV with positive influencing factors (e.g., a robust support system, healthy coping mechanisms) may have greater resilience, resulting in healthier outcomes (e.g., better mental health, disclosure of HIV status). On the other hand, ALHIV with negative influencing factors (e.g., lack of social support, negative attitude) may lack resilience, contributing to stigma-related outcomes (e.g., poor ART adherence, social isolation).

Few prior studies have examined the relationship between individual resilience attributes such as motivation and optimism in the context of ALHIV. Research among adolescents in the US has shown that a positive outlook may moderate the effectiveness of HIV/STI prevention interventions targeting this age group [26]. In addition, a study among Black South African ALHIV found that protective factors at the individual level (e.g., access to information about HIV, self-care) promoted resilience and psychological well-being [27]. Similarly, our study findings support that interventions aimed at promoting resilience at the individual level have potential to overcome internalized stigma. However, the resilience literature provides limited examples of evidence-based interventions targeting ALHIV [28, 29]. Examples include the Collaborative HIV Prevention and Adolescent Mental Health Program which is a family-focused program targeting 9–13-year-olds with HIV [28]. In addition, Dow et al. [29] found that their mental health intervention (Sauti ya Vijana) promoted resilience by addressing internalized stigma, leading to positive behavior change in an individually randomized group treatment study with HIV-positive Tanzanian youth aged 12–24 [29]. Similar interventions with both individual and group sessions may successfully promote resilience among ALHIV in Kenya.

At the interpersonal level, a breadth of research conducted in sub-Saharan Africa shows that disclosure to trusted friends and family members improves outcomes among ALHIV [30–33]. Furthermore, several studies have found that disclosure leads to enhanced social support, which can bolster individual-level resilience factors, including self-efficacy [31, 33]. Qualitative studies with ALHIV in Botswana and Tanzania found that disclosure enabled adolescents to resist HIV stigma and better engage in treatment support [31]. Similarly, a study among ALHIV in Uganda and Kenya found that disclosure was linked to positive individual factors such as self-confidence and personal motivation [33]. Prior research supports that interventions should focus on adolescents’ desire for peer acceptance and social connection by teaching ALHIV how best to disclose to trusted friends and family members and navigate social scenarios [7]. To better address interpersonal stigma, interventions should engage adolescents and individuals in their support systems to address misinformation and stigma and enhance psychosocial and adherence support [34]. While organizations such as LVCT Health often engage HIV-positive and HIV-negative individuals in programming, outreach to a broader audience of caregivers, peers, and school staff may be needed to reduce HIV-related community stigma and improve outcomes.

Although many studies have focused on schools as potential intervention sites for HIV prevention, testing, and education [35–37], few studies have focused on how ALHIV can build resilience and enlist support in school settings. Several PhotoVoice studies conducted with ALHIV in schools support our findings that ALHIV can develop coping strategies in schools [38, 39], highlighting the critical role of friends as resources [40]. Similar to adolescents in our study, ALHIV in other studies have perceived teachers as insensitive, strict, and unsupportive. Training school staff and relaxing attendance policies may enhance understanding, increase inclusivity, and improve clinic attendance among students living with HIV. One such example is the Red Carpet intervention in Homa Bay, Kenya, which has utilized peer-led services including peer counseling, psychosocial support, and health education in schools and health centers to combat stigma for newly diagnosed ALHIV aged 15–21 [40]. This program demonstrated that HCWs, teachers, and other school staff could become meaningfully involved in supporting students with HIV when provided the appropriate training and resources. Furthermore, internal policy changes in schools, such as implementing health education curriculums and anti-stigma campaigns, were shown to improve HIV outcomes (indicated by linkage to care and retention in ART treatment) among ALHIV [40].

Finally, our findings aligned with current research [41, 42], and highlighted that clinic-based programming has the potential to build resilience among ALHIV. Health clinics can support ALHIV in various ways including helping them gain knowledge of SRH resources [25] and teaching strategies for disclosure and interpersonal skill-building [43]. One study conducted with a small sample of ALHIV and adults in the US demonstrated that providers who believed in the inherent strength of their patients were better able to foster resilience among patients [44]. Our findings suggest that adolescents feel more comfortable with HCWs who are warm, non-judgmental, provide comprehensive information, and build trust with patients. Interventions that support HCWs to gain skills necessary to build positive relationships, that foster resilience and promote continued adherence, have potential to improve ALHIV outcomes.

Various forms of peer support programs have effectively improved ALHIV health outcomes through promoting adherence [45]. One systematic review of ALHIV interventions [46] found that several studies that involved peer-delivered mental health interventions resulted in increased ART adherence [29, 47, 48] highlighting the potential role for peer leaders as drivers of interventions to improve ALHIV ART adherence. While the programs included in this study already utilized peer leader programming, our research highlights the need for increased peer leader training, resources, and compensation. Better support for peer leader programs would allow peer leaders to gain a more holistic view of adolescent experiences and provide formal recognition for their key role in supporting ALHIV. Our research highlighted that peer leaders could have a role in supporting adolescents in multiple contexts even outside the traditional clinic setting. These settings include homes and schools, if feasible given school policies and constraints. Peer leaders felt that they could better reach adolescents struggling with adherence through home visits and interaction with adolescent support systems, such as family, school staff, and school peers. In addition, peer leaders living with HIV can be encouraged to disclose their status to ALHIV to serve as role models and provide examples of how they overcame stigma and other challenges themselves.

This study had several limitations. Most participants were in school, and ALHIV, who had dropped out of or completed school, were under-represented. Therefore, researchers did not gain insight into experiences of adolescents in the workplace or those in the community who were unemployed and out of school. Additionally, self-presentation bias among ALHIV may have caused ALHIV participants to focus disproportionately on positive stories, leading to overestimating their resilience. The majority of adolescents sampled were perinatally infected, and perspectives of ALHIV infected through sexual transmission, or injection drug use may not be as well captured. FGDs conducted among AIC and peer leaders included adolescents regardless of HIV status, which may have limited contributions of certain participants (especially individuals living with HIV). While stratifying focus groups by sex was intended to increase participant comfortability surrounding discussion of SRH themes, we recognize that ‘sex’ was defined as binary males and females. This definition excludes nonbinary individuals or sexual and gender minorities. Although no participants disclosed their identity as sexual or gender minorities, the separation of participants along binary sex lines may have discouraged participation from these individuals. Because IDIs and FGDs were conducted in three different languages (including English) while data analysis and manuscript writing was conducted solely in English, there may be language or cultural nuances that have been lost in translation. Our analysis was focused on the experiences of adolescents and young adults. Future analysis of parent/caregiver or HCW perspectives could add additional insight into stigma and resilience experiences.

## Conclusions

Our study used a qualitative approach to capture the personal experiences and perspectives of three AYA participant groups in order to characterize stigma experiences at multiple levels of the socio-ecological model and identify the role of resilience in overcoming stigma barriers. Overall, our study demonstrates the resilience of ALHIV despite facing multiple challenges, and developed a conceptual model that integrates our findings on how HIV-related stigma in four critical contexts combines to impact ALHIV. Our findings suggest that ALHIV can actively cope with challenging social circumstances through individual resilience, social support from family and friends, and strategic supports in school and clinic settings. This study highlights the need for strengths-based interventions that consider the individual, home, school, and healthcare contexts, as well as expanded training of peer leaders, school staff, and HCWs to bolster adolescent support and enhance HIV adherence.

## Supplementary Information

**Additional file 1.**

**Additional file 2.**

**Additional file 3.**

**Additional file 4.**

## Data Availability

De-identified transcripts and the codebook used for analysis will be deposited in the Harvard Dataverse (https://dataverse.harvard.edu/) upon manuscript publication.

## Abbreviations

AIC: Adolescents in the community
ALHIV: Adolescents living with HIV
ART: Antiretroviral treatment
AYA: Adolescents and young adults
FGD: Focus group discussion
HCW: Health care worker
HIV: Human immunodeficiency virus
IDI: In-depth interview
LMIC: Low- and middle-income country
SRH: Sexual and reproductive health
