# Supplementary material for: “Your status cannot hinder you”: the importance of resilience among adolescents engaged in HIV care in Kenya
Source: BMC Public Health. 2022 Jun 30;22:1272. doi: 10.1186/s12889-022-13677-w (PMC9245269; doi:10.1186/s12889-022-13677-w)
Supplement: Supplementary file 1 — Additional file 1. [file 12889_2022_13677_MOESM1_ESM.docx]

**Title: Peer leaders FGD guide- English**

**Target Population: Peer leaders**

1. What roles do you play as a peer leaders for adolescents?
2. What are some of the factors that contribute to adolescents getting HIV infection?

*Probe for: What groups of adolescents are most affected by HIV? (Probe: why are these groups the most affected by HIV?*

1. What information do you think adolescents need on HIV?

*Probe for*

- *Do adolescents discuss their HIV related concerns with you? If Yes what questions do they ask?*
- *What misconceptions do they have in regard to HIV testing and care and treatment?*
- *Who do you think should address each concern mentioned?*
- *What would be the best way of reaching many adolescents with messages on HIV? (Probe specific print, radio, social media, community meetings and events etc.*

1. Where do adolescents in your community go for HIV testing services

Probe for:

- - *Have adolescents discussed any concerns about any of the places mentioned?*
  - *If yes what concerns have been reported?*
  - *What was done about each concern mentioned?*
  - *How can testing services for adolescents be improved?*
  - *Do adolescents have fears about testing for HIV? If Yes what are some of the fears that adolescents have?)*

1. How are adolescents living with HIV/AIDS handled in this community? (Probe: are there instances when you feel they are not accorded equal treatment to those who are not infected?)
2. How does the way adolescents living with HIV are handled in the community affect them? (Probe effects on HTS, HIV care and treatment, disclosure or general service seeking).
3. Do you play any role in ensuring that adolescents who test HIV positive are enrolled in care? Please explain your answer

*Probe for*

- *What are the concerns of HIV positive adolescents while deciding where/ whether to enrol for care*
- *What are some of the reasons why some adolescents delay in taking ARVs?*
- *What challenges do you face in ensuring adolescents are enrolled in care?*
- *How can we encourage more HIV positive adolescents to enrol for care?*

***(Anti-retroviral drugs (ARVs) refer to a combination of HIV medicines that are taken daily to treat HIV infection.)***

1. Some HIV positive adolescents refuse taking up HIV medication. What are some of the reasons for this?
2. What concerns do adolescents have with HIV medication?
3. Where do adolescents prefer to take their HIV medications? (Probe for reasons for preference?).

*Probes:*

- *Have adolescents mentioned any concerns they have with HIV services in any of the health facilities mentioned?*
- *What has been done about these concerns and by whom?*
- *How can HIV care services for adolescents be improved?*

1. What challenges do you encounter in encouraging adolescents to test for HIV?
2. What challenges do you encounter encouraging adolescents to take HIV medication as scheduled?

*Probe for have you received any support to deal with the challenges mentioned? Please explain.*

1. What more can be done to support you to effectively support adolescents to take up HIV services (testing and care and treatment)

**Thank you for your time and participation**
