# Supplementary material for: “Your status cannot hinder you”: the importance of resilience among adolescents engaged in HIV care in Kenya
Source: BMC Public Health. 2022 Jun 30;22:1272. doi: 10.1186/s12889-022-13677-w (PMC9245269; doi:10.1186/s12889-022-13677-w)
Supplement: Supplementary file 2 — Additional file 2. [file 12889_2022_13677_MOESM2_ESM.docx]

**Title: ALHIV IDI guide- English**

**Target Population: ALHIV**

1. Please tell me about yourself; what would you like to be or do in future?
2. Please share with me how you found out about your HIV status?

Probe for:

- At what age did you get to know your HIV status?
- How did you find out?
- ***Ask only where adolescent was born with HIV:*** How was disclosure done, who did it, how has this affected your life?
  - *What worked well in the process of finding out your HIV status*
  - *What did not work well in the process of finding out your HIV status*
  - *What can be done differently to make that process easier for young people like yourself?*

1. Are there people to whom you have disclosed your HIV status?
   - *What are the reasons for disclosing your HIV status to the people you have mentioned (explore each person)*
   - *For every person mentioned please explain what made it easy or hard to share with them your HIV status?*
   - *Did you receive any help to disclose your HIV status to the people mentioned? If yes from whom?*

**Ask question 4-5 to adolescents who found out their HIV status through testing. Skip for those born with HIV)**

1. How long did it take you from the time of finding out your HIV status to when you enrolled at the clinic in this facility?
   - *Was it easy for you to enrol at the clinic? Please explain your answer*
   - *Did you receive any support to help you enrol at the clinic? If yes what support and from whom?*
   - *What can we do to make it easier for adolescents to come to our HIV clinic after testing HIV positive?*
2. Are there people who discouraged you from enrolling in the HIV clinic? Please explain your answer

*Probe if yes how did each person mentioned discourage you from enrolling in the HIV clinic.*

***We need to understand how people with HIV are really doing with their pills. Please tell us what you are actually doing. We need to know what is really happening, not what you think we “want to hear.” You will not be punished for any information that you share with us and we will not mention what you say to your parents or health provider.***

1. What is your experience with taking ARVs? Probe for what has worked and what has not worked well?

- *Please tell me your experience taking pills at home*
- *Please tell me your experience taking pills in school*.

1. Please tell me your HIV drugs routine.

- *Are there times you have found it difficult to take your treatment as scheduled? If yes please explain the reasons.*
- *Are there times that you have found it easy to stick to your drug routine? If yes what made it easy?*

1. Who has been the most supportive person to you in ensuring that you remember to take your HIV medication?
2. Do you receive any support from family in remembering to take your medication? Please explain your answer?

*Probe for:*

- *If yes what support has been received?*
- *Are you satisfied with the support?*
- *What more can family do to support you in remembering to take your medication?*

1. Have you disclosed your HIV status to any of your friends? Please explain your answer

Probe for

- *If yes are you receiving any support from your friends to make it easier to take HIV medication?*
- *Are you satisfied with the support?*
- *What more can friends do to support you in remembering to take your medication?*

1. **Skip question 11 if adolescent is not in school**: Have you disclosed your HIV status to any of your teachers? Please explain your answer

*Probe for*

- *If yes are you receiving any support from your teachers to make it easier to take medication while in school?*
- *Are you satisfied with the support?*
- *What more can teachers do to support you in remembering to take your medication?*

1. Are you receiving any support from your health provider to make it easier to take medication as scheduled?

*Probe for*

- *If yes what support?*
- *Are you satisfied with support offered?*
- *What more can they do*

**Clinic appointment**

1. What is your experience with appointments at this health facility?

Probe for

- - *How often are you required to visit the clinic?*
  - *Are there times that you have found it difficult to visit the health facility as scheduled? If yes/ no please explain the reasons.*
  - *What would make it easier for you to attend all scheduled visits?*
  - *Who has been the most supportive person in ensuring that you remember to make your clinic visit as scheduled? Please explain your answer*

1. Are you receiving any support from your health provider to make it easier to remember and keep your clinic visits?

*Probe for*

- - *If yes what support?*
  - *Are you satisfied with support offered?*
  - *What more can they do*

1. *What support do you need to ensure that you attend all your clinic visits?*
   - Support from friends in remembering to make clinic visits as scheduled.

**(Where child has not disclosed status to friends ask about what friends can do to make it easier for them to remember and attend clinic visits and skip to next question)**

Probe for

- *Have you receiving any support from your friends to make it easier to remember clinic visits?*
- *Are you satisfied with the support?*
- *What more can friends do to support you in remembering to take your medication?*

1. Support from teachers (**Skip question if adolescent is not in school)**

**Where adolescent has not disclosed HIV status to any teacher ask about the kind of support they need from teachers to make it easier to remember and attend clinic visits and skip to next question)**

*Probe for*

- *If yes are you receiving any support from your teachers to make it easier to take remember and keep clinic visits while in school?*
- *Are you satisfied with the support?*
- *What more can teachers do to make it easier to remember and keep clinic visits?*

1. If you were given a chance to change one thing at the HIV clinic what would it be?
