# Supplementary material for: “Your status cannot hinder you”: the importance of resilience among adolescents engaged in HIV care in Kenya
Source: BMC Public Health. 2022 Jun 30;22:1272. doi: 10.1186/s12889-022-13677-w (PMC9245269; doi:10.1186/s12889-022-13677-w)
Supplement: Supplementary file 3 — Additional file 3. [file 12889_2022_13677_MOESM3_ESM.docx]

**Title: Adolescent FGD guide- English**

**Target population: Adolescent in the community**

**Now I am going to ask you questions about HIV/AIDS and sexual health? We would like you to tell us about your views and those of other adolescents (10-19yrs) in your community.**

- 1. How do adolescents protect themselves from getting HIV?
  2. What information do you think adolescents need on HIV?
  3. What are some of the reasons that make adolescent **(specify according to sex of participants - boys or girls)** vulnerable to HIV infection?

*Probe for behaviours that put adolescents at risk of HIV infection*

- 1. What are some of the reasons that make adolescents go for a HIV test?
  2. Where do most adolescents prefer to go for HIV testing services from:

*Probe for reasons for each place mentioned*

*Probe for challenges experienced in getting tested in mentioned places*

- 1. Are there other places where adolescents would prefer to get tested for HIV?

*Probe for other testing venues that can be used to reach adolescents and reasons for each place mentioned*

- 1. Are there places where adolescents would not like to be tested?

*Probe: reasons for each place mentioned*

- 1. Do adolescents have fears about testing for HIV?

*Probe if yes what are some of the fears that adolescents have about HIV testing?*

- 1. How are adolescents living with HIV/AIDS handled in this community?

*Probe: are there instances when you feel they are not accorded equal treatment to those who are not infected?*

*Probe how does the way adolescents living with HIV are handled in the community affect them?*

- 1. How are adolescents living with HIV/AIDS handled in the school?

*Probe how does the way adolescents living with HIV are handled in the community affect them?*

**I would like us to discuss HIV Care and Treatment services for adolescents. Please give us your honest views. The information you share will not be traced back to any individual.**

- 1. What are your views about HIV treatment (ARV’s) for adolescents who are HIV positive?

Probe for what they know/have heard about HIV treatment/ ARV’s

- 1. Some HIV positive adolescents either delay or refuse taking up HIV medications. What are some of the reasons for this?
  2. Where would adolescents prefer to take their HIV medications? (Probe in nearest health facility? Or facility further away? Public/ private facility? Reasons for preference?).
  3. What can encourage adolescents who are positive to enrol for HIV treatment services?
  4. If we wanted to get many adolescents to come for HIV services, who in your community would be the most influential in helping us to encourage adolescents to come for HIV services?

**Now I would like us to discuss Sexual and Reproductive Health Services for adolescents**

- 1. What comes to mind when you hear of Sexual and Reproductive health services

Probe Do you know of health problems that result from unwanted or unprotected sexual activity? Do any of these affect adolescents in this area?”)

- 1. What do adolescents like yourselves do when affected by the problems you have mentioned?

*If they have mentioned SRH problems like unwanted pregnancy or STIs, ask further questions such as:*

- *If someone your age wanted to get some condoms, where could s/he get them?*
- *If someone your age got an STI, what would they do?*
- *If someone your age thought she was pregnant, what would s/he do?*
- *If someone your age wanted to prevent pregnancy what would she do?*
- *If someone your age had problems with their menses what would she do?*

| \| - *If someone your age was forced to have sex what would s/he do* \| \| --- \| |
| --- | --- |
