# Supplementary material for: “Your status cannot hinder you”: the importance of resilience among adolescents engaged in HIV care in Kenya
Source: BMC Public Health. 2022 Jun 30;22:1272. doi: 10.1186/s12889-022-13677-w (PMC9245269; doi:10.1186/s12889-022-13677-w)
Supplement: Supplementary file 4 — Additional file 4. [file 12889_2022_13677_MOESM4_ESM.docx]

**Title: Adolescent FGD guide- English**

**Target Population: ALHIV**

**Now I am going to ask you questions about HIV/AIDS and sexual health? We would like you to tell us about your views and those of other adolescents (10-19yrs) in your community.**

- 1. What are some of the factors that contribute to adolescents getting HIV infection?
  2. What information do you think adolescents need on HIV and Sexual and Reproductive health?
  3. Where do adolescents prefer to access HIV treatment services from: (Probe for reasons).
  4. If we wanted to get many adolescents to come for HIV services, who in your community would be the most influential in helping us to encourage adolescents to come?
  5. How are adolescents living with HIV/AIDS handled in the community? (Probe: are there instances when you feel they are not accorded equal treatment to those who are not infected?)
     - Probe for how the treatment affects them; effects on HIV care and treatment, disclosure or general service seeking).
  6. How are adolescents living with HIV are treated in school?
     - Probe for how the treatment affects them; effects on HIV care and treatment, disclosure or general service seeking).

**I would like us to discuss HIV Care and Treatment services for adolescents. Please give us your honest views. The information you share will not be traced back to any individual.**

- 1. What are your views about HIV treatment (ARV’s) for adolescents who are HIV positive?
  2. Some HIV positive adolescents either delay or refuse taking up HIV medications. What are some of the reasons for this? (probe for in school. At home)
  3. What are some of the reasons why some adolescents find it hard to take their medication as scheduled
     - *Probe for (In school? At home during holidays)*
     - *Probe for what can be done to address these challenges and by whom?*
  4. What are some of the reasons why some adolescents find it hard to keep their clinic appointments?
     - *Probe for what can be done to address these challenges and by whom?*
  5. Where would adolescents prefer to take their HIV medications? Reasons for preference?).
  6. What can encourage more adolescents who are HIV positive to enrol for HIV treatment?
  7. To which types of people do adolescents disclose their HIV status to?

*Probe for:*

- - - *Why it is easy to disclose to the mentioned people*
    - *What are some of the challenges of disclosing to mentioned people*
    - *What more can be done to address the challenges mentioned.*
    - *What kind of support do mentioned people give.*
  1. What are your suggestions for improving HIV services to adolescents? Probe for if you were given a chance to change one thing at the clinic in (mention name of facility) what would it be?
